# Improving quality for maternal care - a case study from Kerala, India

**DOI:** 10.12688/f1000research.7893.1

**Published:** 2016-02-12

**Authors:** Ioana Vlad, VP Paily, Rajeev Sadanandan, Françoise Cluzeau, M Beena, Rajasekharan Nair, Emma Newbatt, Sujit Ghosh, K Sandeep, Kalipso Chalkidou

**Affiliations:** 1 London School of Hygiene and Tropical Medicine, London, UK; 2 Department of Obstetrics and Gynaecology, Rajagiri Hospital, Kerala, India; 3 Central Secretariat, Ministry of Rural Development, Government of India, New Delhi, India; 4 NICE International, National Institute for Health and Care Excellence, London, UK; 5 Government of Kerala, Kerala, India; 6 Department of Obstetrics and Gynaecology, S.U.T. Hospital, Kerala, India; 7 ITAD , Hove, UK; 8 Monitoring and Evaluation, National Health Mission, Kerala, India

**Keywords:** maternal health, quality improvement, evidence-informed policy-making

## Abstract

Background: The implementation of maternal health guidelines remains unsatisfactory, even for simple, well established interventions. In settings where most births occur in health facilities, as is the case in Kerala, India, preventing maternal mortality is linked to quality of care improvements.

Context: Evidence-informed quality standards (QS), including quality statements and measurable structure and process indicators, are one innovative way of tackling the guideline implementation gap. Having adopted a zero tolerance policy to maternal deaths, the Government of Kerala worked in partnership with the Kerala Federation of Obstetricians & Gynaecologists (KFOG) and NICE International to select the clinical topic, develop and initiate implementation of the first clinical QS for reducing maternal mortality in the state.

Description of practice: The NICE QS development framework was adapted to the Kerala context, with local ownership being a key principle. Locally generated evidence identified post-partum haemorrhage as the leading cause of maternal death, and as the key priority for the QS. A multidisciplinary group (including policy-makers, gynaecologists and obstetricians, nurses and administrators) was established. Multi-stakeholder workshops convened by the group ensured that the statements, derived from global and local guidelines, and their corresponding indicators were relevant and acceptable to clinicians and policy-makers in Kerala. Furthermore, it helped identify practical methods for implementing the standards and monitoring outcomes.

Lessons learned: An independent evaluation of the project highlighted the equal importance of a strong evidence-base and an inclusive development process. There is no one-size-fits-all process for QS development; a principle-based approach might be a better guide for countries to adapt global evidence to their local context.

## The challenge

For low and middle-income countries (LMIC) moving towards Universal Health Coverage, social protection and the expansion of access to essential health services need to be balanced with efforts to ensure quality of care ^1^. In the case of maternal health, addressing the “quality care gap” for births occurring in health facilities is central to achieve the promised reductions in maternal deaths by implementing the available effective and cost-effective interventions ^2^. However, implementation of global clinical guidelines remains unsatisfactory, even in the case of basic, well established interventions ^3, 4^. In this context, there have been calls for strengthening the evidence base on the implementation of such interventions in LMIC settings, including in terms of feasibility and replicability ^5^.

This paper describes the partnership between NICE International, a division of the National Institute for Health and Care Excellence (NICE), United Kingdom, the Government of Kerala, India, and the Kerala Federation of Obstetrics and Gynaecology (KFOG) in developing and implementing the first clinical quality standards (QS) for reducing maternal mortality in the state, based on robust clinical guidelines. It thus explores the complex process of developing and implementing evidence-based QS in a LMIC setting and, more significantly, the difficulty of embedding QS in quality improvement processes within the health system.

Quality standards are a concise sets of prioritised statements designed to drive measurable quality improvements ^6^. They provide “health policymakers, health insurers, service providers, healthcare professionals and patients with definitions of what high quality healthcare looks like in practice; and related performance measures that are reliable and meaningful to the local setting in which they are used” ^7^. As such, the development and implementation of QS are highly context-specific. By presenting the process of developing and implementing QS in a LMIC context, this paper highlights lessons learned for similar settings, while acknowledging that the process cannot be simply transferred from setting to setting. In doing so, the paper also reflects on global health partnerships and on the sustainability of evidence-informed decision-making processes established through such partnerships ^8^. Box 1 summarises the key messages of this paper.

Box 1. **Summary points**

1. In the development of QS in LMIC contexts, local buy-in for the process of development as well as for the end product bear equal importance.
2. There is no one-size-fits-all process for QS development - instead, a principle-based approach might be a better guide for countries to adapt global evidence to their local context.
3. Agencies like NICE International, which provide technical support for evidence-informed decision-making processes, will maximise their effectiveness if the local demand and the capacity for such processes (and their products) is stimulated and policy makers fully engaged.

## Background to the partnership

NICE International started engaging with the Indian state of Kerala in 2009, at the invitation of the then Minister and Principal Secretary for Health, with a workshop organised by the state’s Ministry of Health. The workshop was designed as a multidisciplinary consultation around priority areas for the Kerala Ministry of Health, related to quality of care: quality standards and standard operating procedures for hospital providers; standard treatment guidelines (STGs); and approaches to implementation of STGs and clinical pathways. What followed was an exploratory period including several visits to NICE’s headquarters by Kerala policy-makers and characterised by the creation of informal networks with individuals in leadership positions from government bodies and professional organisations in Kerala. This period of informal engagement culminated in a project conceived to support the Government of Kerala in the development and implementation of QS for preventing maternal mortality, a policy priority for the state.

Although Kerala has the lowest maternal mortality rate (MMR) in India and has achieved the associated Millennium Development Goal targets ^9^, its government had pledged to reduce it further, aiming at a 50% decrease in MMR by 2017, the end of its 12 ^th^ five year plan on medical and public health ^10^. Table 1 outlines key indicators related to maternal health in Kerala, by comparison to the national level.

**Table 1.** Maternal health landscape in Kerala.

	Kerala	India
**Total Population**	33.4 Million	1.237 Billion
**Infant Mortality Rate**	12	42
Maternal Mortality Ratio *	66	190
**Fertility Rate ^§^**	1.6	2.5
**Female Literacy (%)**	91.98	65.4

*2010–12 Sample Registration Survey ^§^The World Bank, 2013. WHO, UNICEF, UNFPA, The World Bank, & UN Population Division Maternal Mortality Estimation Inter-Agency Group; **modelled estimate,** **2014**.

The priority given to maternal health by the Government can be explained by the fact that, compared to other health indicators, such as life expectancy and infant mortality rate, the MMR had remained relatively high, with no significant improvements in recent years. Ninety eight per cent of deliveries in Kerala take place in institutions, suggesting that efforts to reduce avoidable maternal deaths should focus on improving care at health facilities ^11^. Out of the over 500 000 deliveries registered in Kerala annually, approximately 72% take place in private facilities, and 28% in public government teaching and non-teaching hospitals ^12^. The high rates of institutional delivery, low fertility and high female literacy suggested that reductions in avoidable maternal deaths are possible through improvements in quality of care.

## Methods

### Development of quality standards in Kerala

The formal partnership between the Government of Kerala, represented by the Principal Secretary for Health and Family Welfare, the KFOG and NICE International was launched in 2012. It resulted in ten QS related to management and prevention of post-partum haemorrhage (PPH) (QS 1-5) and hypertension in pregnancy (QS 6-10). The QS were launched in January 2013, in the presence of the UK Health Minister and the Principal for Health in Kerala.

Local ownership was a core principle of the QS development process, with the Government of Kerala and KFOG taking a leadership role. NICE International’s contribution was to (a) provide a technical and methodological framework for the development of the QS; (b) support the institutional partnerships between the Government of Kerala, the KFOG and other local stakeholders.

In terms of technical and methodological support, the NICE framework and processes that underpin development of QS for the UK National Health Service (NHS) were used. This includes the selection of high-priority topics for quality improvement in a defined service area, and combines evidence-based guidance with stakeholder priorities and evidence of current practices through a deliberative process (ran by a QS advisory committee), ultimately resulting in a series of quality statements and corresponding measurable indicators ^13^. For the NHS, as for the Kerala health system, developing QS is a highly contextualised process. As such, the NICE framework was adapted to fit the local context, by identifying institutional decision-making rules, key stakeholders and health system functioning characteristics in Kerala.

**Figure 1.**
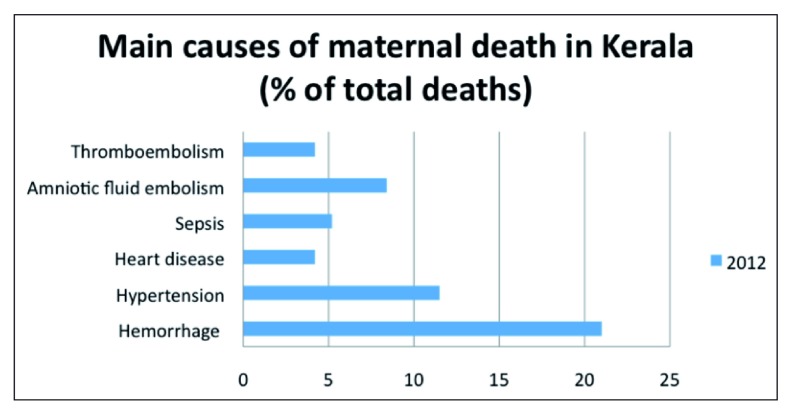
Main causes of maternal deaths in Kerala, 2012. Adapted from the 2 ^nd^ edition of the CRMD ^12^.

Locally generated evidence played a key role, particularly in the identification of the leading causes of maternal death in Kerala and therefore the key priorities for the QS. The process was fundamentally informed by the Confidential Review of Maternal Death (CRMD) Audit, which has been run by the KFOG since 2004 and is the only one of its kind in India ^11, 12^. As shown in Figure 1, PPH and hypertension are the two main causes of maternal mortality. Furthermore, they are estimated to consistently account for between 29–44% of maternal deaths in Kerala between 2006–2009 ^14^.

Together with the findings of the CRMD, local epidemiological data and routinely reported systems data (e.g., the Sample Registration System conducted by the Office of the Register General) were assessed using deliberative multi-stakeholder processes convened with mentorship from NICE International. This mentorship consisted of a total of eight workshops (four during the development period and four during implementation). The multi-stakeholder workshops that comprised the QS development process functioned to support the creation and maintenance of institutional links, most notably between KFOG and the state’s Government. Following an initial workshop in June 2012, focused on the active management of the third stage of labour (one of the ten statements included in the first edition of the finalised standards), a multidisciplinary group was established to lead on the development of the standards, the wider consultation process for each statement, and the implementation of the QS. Members included key policy-makers (the Principal Secretary, the Director of the State's National Health Mission-NHM) and leading gynaecologists and obstetricians, nurses and administrators from across Kerala.

The group developed quality statements derived from evidence-based guidelines published by the KFOG, NICE, the World Health Organization, and the UK Royal College of Obstetricians and Gynaecologists (see Box 2 for an example of a quality statement and its link to global guidelines), but adapted based on the experience of practising obstetricians and nurses in Kerala.

Box 2. **Example of guideline recommendations and their adaptation in the Kerala QS. Adapted from *Principles for developing clinical quality standards in low and middle income countries* (14)**

**Table:** 

Contextualization of global evidence – Global guidelines and Kerala QS for active management of third stage of labour	
**WHO recommendation**	● The use of uterotonics for the prevention of PPH during the third stage of labour is recommended for all births. ● Oxytocin (10 IU, IV/IM) is the recommended uterotonic drug for the prevention of PPH. ● If intravenous oxytocin is unavailable, or if the bleeding does not respond to oxytocin, the use of intravenous ergometrine, oxytocin-ergometrine fixed dose, or a prostaglandin drug (including sublingual misoprostol, 800 μg) is recommended.
**Kerala Quality Standard**
	**Quality statement**
	Women who have given birth either vaginally or by caesarean are offered a bolus dose of Oxytocin, Ergometrine or Protaglandin F2 Alfa at the time of delivery of the shoulder or within 1 minute of the delivery of foetus to prevent post-partum haemorrhage and to assist delivery of the placenta.
	**Definitions**
	Definitions of, e.g., “third stage of labour”, “active management of third stage of labour”, “Oxytocin”.
	**Quality Measure**
	**Structure:** a) Evidence of agreed guidelines or protocols in the hospital for the active management of the third stage of labour. b) Display of flow charts based on agreed guidelines, protocols or clinical pathways in the labour room. c) Evidence of availability of Oxytocin, Ergometrine and PG F2 Alfa at the place of delivery. d) Evidence of suitable storage facilities (refrigerator) for the drugs. e) Evidence of equipment for measuring blood loss. **Example of process measure for vaginal deliveries:** Proportion of women giving birth vaginally who receive the Oxytocin, Ergometrine or PGF2 Alfa during third stage management of labour during the month (including numerator/denominator indicators). **Example of outcome measure for vaginal deliveries:** Proportion of women who experience an estimated blood loss equal to or more than 500 ml during and or following a vaginal delivery (including numerator/denominator indicators).
	**Explanations of what the QS means for each audience**
	Service providers, healthcare professionals, payers.
	**Data Sources**
	Data collection needs and procedures for the monitoring of the QS implementation (e.g., labour room register, monthly reporting forms to the NHM).
	**Source Guidance**
	Sources of global and local evidence used for the development of the statements and of the indicators.

### Pilot implementation of the Quality Standards

The QS implementation required action ranging from monitoring (antenatal and labour), to administering drugs and blood products, to referral procedures. These actions raised implementation needs related to data collection mechanisms, on the one hand, and state-wide implementation support mechanisms, on the other. Through the consultation processes, the multi-stakeholder group identified practical methods for implementing the standards and monitoring outcomes. The implementation plan, including an initial needs assessment, identified the need for staff training, as well as for upfront investment in infrastructure, improvements of drug availability and the need to support clinical audit. All 400 staff working in the maternity wards of the pilot health facilities participated in an initial training carried out by the KFOG. The Government of Kerala allocated the funds needed to cover these needs for the pilot period, as per the core tasks outlined in Box 3.

Box 3. **Core tasks for the implementation of the QS**

**Table:** 

Orientation meeting	An information meeting held by the NHM director with directors of pilot hospitals to explain the QS work.
Needs assessment of pilot hospitals	Each pilot hospital conducted an inventory of equipment, staffing and other components needed for the QS implementation to identify gaps in existing resources.
Baseline data collection forms	Data collection proforma to collect retrospective baseline data on QS indicators in pilot hospitals.
Reporting form and registers - design and printing	Redesigning existing labour registers to collect data for QS indicators.
QS document	Edit, design, print and launch of the QS document.
Flow charts design and printing	Posters representing the QS to be posted in labour wards.
Training	2-day training sessions for all frontline maternity staff (400) in the pilot hospitals.
Human Resources	Staff redeployment needed to ensure adequate capacity to implement QS (e.g., 2-hour observation post-delivery).
Procurement	Procurement of equipment, materials and drugs needed.

Implementation pilots started in six public and two private maternity hospitals across Kerala in April 2013. In line with the plan, pilot hospitals collected monthly data on the QS indictors from their delivery registers and sent them to the NHM for analysis. These data were discussed in monthly review meetings chaired by the NHM Director, where the staff from the pilot maternities, members from the KFOG and technical staff from the NHM reviewed progress reports and provided feedback on the process of implementing the standards. These meetings were designed to create a feedback loop supporting implementation.

## Results

### Lessons learned

An independent, qualitative evaluation of NICE International’s engagement in Kerala was conducted in order to shed light on the lessons learned following a year of QS implementation (2013–2014). Drawing on key informant interviews and a document review, the assessment focused on participants’ perception on the overall value of the QS, as well as how the staff training helped implement the QS and improve practice. The evaluation also examined the extent to which similar initiatives had been taken up in other locations and whether new, similar partnerships were developed by NICE International in India. The focus on perceptions about the process highlights the importance placed by NICE International on the local ownership of the project ^15^.

With regards to the overall value of the QS, participants valued the deliberative development process as being critical in producing statements and indicators that were relevant and acceptable to both clinicians and policy-makers in Kerala. This deliberative approach, which participants indicated was innovative for producing QS in Kerala, was seen as having maximised the likelihood of implementation, especially given that the major partners in developing the QS were also involved in piloting. There was particular value placed on the fact that NICE International’s approach is non-prescriptive, thus allowing for the QS development and implementation to be locally owned and driven by Kerala institutions. It thus resulted in guidance that was perceived as detailed and explicit, compared to other more general sources of guidance. However, despite a high buy-in for the implementation process, there remained a perceived tension between providing standardised care and professional freedom. Consequently, it remains to be seen whether similar level of acceptance would be replicated when the QS are scaled up across Kerala, when it is expected that the main value of the QS would stem from their perceived role in improving quality of care and/or clinical outcome.

With regards to implementation, the QS were perceived as a valuable tool to improve and standardise quality of care, exemplified by changes in practice such as: the introduction of management of fourth stage of labour; the use of sterile delivery kits; greater consistency in the management of the third stage of labour and the use of oxytocin; the measurement of blood loss instead of subjective estimation; and better record keeping. Furthermore, staff from the sampled pilot maternities reported high satisfaction with the QS and in some cases increased confidence in PPH management when following the standards.

Challenges referred to insufficient staffing in some of the pilot hospitals, on the one hand, and variability in practice, on the other. Specifically, the independent evaluation suggested a lack of sufficient staff for managing fourth stage of labour and variations in some of the actions indicated by the QS, i.e., use of magnesium sulphate to prevent eclampsia and of urine protein testing. This highlights the importance of standard operating procedures for each facility, which need to be updated during and beyond the piloting phase. These standard operation procedures refer to the need that each facility consider the QS implementation in term of staff training needs, drug supply, referral systems and staffing for the needed monitoring ^14^. Furthermore, the importance of staff training was highlighted as key for the sustainability of the implementation, particularly given the observed high turnover of labour room staff. Pilot data suggested that a refresher of the initial staff training provided by KFOG is needed in order to respond to the requirements of implementation and ensure its sustainability. Plans for ongoing training, under discussion at several review meetings, highlighted the need to target both central level staff (to ensure continued support for the implementation), as well as clinicians and nurses (to account for staff turnover and as a refresher).

Another important lesson learned stems from the lack of baseline data (which was discussed at the workshops, but not collected during implementation), as it would have allowed a quantitative assessment of quality improvements. A related issue was that the compilation of data in the maternity wards and the feedback loop between the NHM and the pilot maternities has not functioned as well as was expected. For example, the data recorded in the labour registers designed for implementing the QS were not analysed systematically in order to identify needed adjustment to the implementation. In addition, few facilities carried out clinical audit, which would have provided an opportunity for staff to reflect on their practice. As a result, the monthly meetings between the NHM and facilities identified deficiencies in monitoring improvement in the process of care. This highlights the need to strengthen local data analysis capacity, as well as ongoing staff training throughout the implementation of the QS, whether at pilot level or for scale up.

### The road ahead

The Kerala experience up to this point illustrates a process of engagement with top-level policy-makers that built on global and existing local evidence and led to the implementation of QS at facility level. In recording elements of this experience, this paper provides an account of the process of complex decision-making informed by evidence in a “real life” situation in a LMIC setting, across different institutional levels of the health system, with international support, but limited international funding for implementation.

A major takeaway from this process, which is ongoing, refers to the importance of engaged professional and policy leaders who are able to promote rapid change in the community, with a high level of visibility and acceptability. However, there is a danger that the continuity of the process may be affected when key individuals change posts and responsibilities and their initial input cannot be sustained. NICE International’s engagement in Kerala aimed to support sufficiently robust institutional links for the QS implementation process to continue when unavoidable changes in leadership happened. As a complex health system intervention, future will tell the extent to which this has been successful. It is encouraging that the Government of Kerala decided to expand the implementation of the QS to another 32 health facilities in Kerala. Furthermore, the process for developing the standards has garnered interest at state level and across India. The Government of Kerala completed QS for neonatal care in 2015, recently pledging Rs. 25 crore (USD4 million) for their implementation in 22 hospitals as well as expressing interest in their applications to antenatal care. Combined staff training is now taking place to implement both the maternal and neonatal QS in these facilities.

Similarly, there are nascent plans to draw on the QS development processes in other Indian states and at central level. Government officials in other states (Bihar, Odisha), having visited Kerala and interacted with NICE International, have expressed interest in the CRMD and QS process. Furthermore, the Rashtriya Swasthya Bima Yojna (RSBY), the government health insurance scheme for population under the poverty line, commissioned the development of clinical pathways for seven medical conditions, using principles for evidence-informed decision-making that were at the core of the QS development process, with the goal to eliminate the practice of wasteful and expensive medical procedures. Further, the Ministry of Health and Family Welfare (MoHFW) has commissioned the development of twelve Standard Treatment Guidelines that include Quality Standards for twelve health conditions, using a similar framework to the one used in Kerala.

However, the independent assessment carried out by Itad ^15^ suggests that some stakeholders valued the QS more as a product, rather than as a process that can be scaled up and replicated. This difference in perceptions regarding replicability highlights the importance of local buy-in for the principles of evidence-informed decision-making, and not only for its products (in this case, the QS). The process of participative development needs to be carried out in each specific context. Even if the product turns out to be similar across contexts, local input in the process is too vital to be bypassed, especially with regards to the feasibility of implementation. Recognizing this, NICE International has developed a guide containing principles (and not a standardised process) for developing QS in LMICs, using examples based on the experience in Kerala and suggesting ways of adapting such principles to diverse local contexts ^7^.

Global health partnerships as the one presented here, need to support institutionalised processes of evidence-informed decision-making at local level; this is critical for sustainability, especially when faced with unavoidable changes in leadership. While informal networks can initially help establish such partnerships, this desirable shift from informal to institutional links promotes good governance in global health partnerships ^16^. Organisations like NICE International, which provide technical support for evidence-informed decision-making processes, will maximise their impact if the local demand and the capacity for such processes (and their products) is stimulated and constantly engaged.
