# Supplementary material for: Improving quality for maternal care - a case study from Kerala, India
Source: F1000Res. 2016 Feb 12;5:166. [Version 1] doi: 10.12688/f1000research.7893.1 (PMC4926753; doi:10.12688/f1000research.7893.1)
Supplement: Peer review history

# Peer review: Referee response for version 1

**DOI:** 10.5256/f1000research.8496.r14519

**Published:** 2016-07-06

**Authors:** Rachel Foskett-Tharby

**Affiliations:** 1 National Collaborating Centre for Indicator Development, Primary Care Clinical Sciences, University of Birmingham, Birmingham, UK

This is an interesting report of a quality improvement initiative which highlights the importance of such activities being piloted and refined prior to widespread implementation. I have a couple of minor comments:

1. Firstly, given that the evaluation appeared to cover the full quality standard it would be useful for readers to be able to review the full list of QS statements. Could this be added as a table or supplementary information?
2. Secondly, the first paragraph of the results describes the methods of the evaluation. The authors may wish to move this to methods section and provide more detail.
3. Thirdly, it would be useful to have some more information about the participants in the evaluation e.g. roles, participation in the development of the QS as this would provide some context for the findings.

I have read this submission. I believe that I have an appropriate level of expertise to confirm that it is of an acceptable scientific standard.

# Peer review: Referee response for version 1

**DOI:** 10.5256/f1000research.8496.r14606

**Published:** 2016-06-27

**Authors:** Marleen Temmerman

**Affiliations:** 1 International Centre for Reproductive Health (ICRH), Department of Uro-gynaecology, Ghent University, Ghent, Belgium

Interesting paper addressing an important public health topic. Well written and contains methods and models that could be replicated and implemented in different settings.

I have read this submission. I believe that I have an appropriate level of expertise to confirm that it is of an acceptable scientific standard.

# Peer review: Referee response for version 1

**DOI:** 10.5256/f1000research.8496.r12582

**Published:** 2016-02-22

**Authors:** Nerges Mistry, Shilpa Karvande

**Affiliations:** 1 Director and Trustee, Foundation for Research in Community Health, Mumbai, Maharashtra, India; 2 Research Officer, Foundation for Research in Community Health, Pune, MH, India

The case study by Vlad *et al.* is a significant example how quality initiatives are nested within the public health system of Kerala – a “health iconic” State of South India.

The representativeness of this case study for other States in India (particularly those with weak health systems and poor socio-economic indices) is possibly quite weak and needs to have a cautionary mention in the discussion.

The uniqueness of the components of the Kerala initiative need to be highlighted. In my opinion these would comprise of (a) the significant and dedicated contribution of private participants and the KFOG in persistently facilitating the quality initiatives yet placing it in the hands of the public health sector (b) the wise buy in of the Government of Kerala of international evidence based initiatives and for forging a symbiotic relationship with the private sector (e) facilitation of completion of approach through implementation processes such as definition of quality benchmarks and facility level training.

It is hoped that the pioneering efforts of Kerala find wide resonance in other parts of India

We have read this submission. We believe that we have an appropriate level of expertise to confirm that it is of an acceptable scientific standard.
